# Supplementary material for: MetaQTL: a package of new computational methods for the meta-analysis of QTL mapping experiments
Source: BMC Bioinformatics. 2007 Feb 8;8:49. doi: 10.1186/1471-2105-8-49 (PMC1808479; doi:10.1186/1471-2105-8-49)
Supplement: Additional File 7 — MetaQTL Package : jar file and tutorial. This Zip archive contains both the MetaQTL JAR file and the files of the tutorial. [file 1471-2105-8-49-S7.zip › org.inra.metaqtl/doc/org/metaqtl/graph/class-use/ChromLayer.CMrkTickPoint.html]

Uses of Class org.metaqtl.graph.ChromLayer.CMrkTickPoint


|  |  |  |
| --- | --- | --- |
| **Overview** \| **Package** \| **Class** \| **Use** \| **Tree** \| **Deprecated** \| **Index** \| **Help** | |  |
| PREV   NEXT | **FRAMES**    **NO FRAMES**     **All Classes** |  |


---


## **Uses of Class org.metaqtl.graph.ChromLayer.CMrkTickPoint**

| Packages that use ChromLayer.CMrkTickPoint | |
| --- | --- |
| **org.metaqtl.graph** |  |

Uses of ChromLayer.CMrkTickPoint in org.metaqtl.graph

| Methods in org.metaqtl.graph that return ChromLayer.CMrkTickPoint | |
| --- | --- |
| `ChromLayer.CMrkTickPoint[]` | `ChromLayer.getCommonMarkerPoints(ChromLayer chromLayer)` |

---


|  |  |  |
| --- | --- | --- |
| **Overview** \| **Package** \| **Class** \| **Use** \| **Tree** \| **Deprecated** \| **Index** \| **Help** | |  |
| PREV   NEXT | **FRAMES**    **NO FRAMES**     **All Classes** |  |


---
